# Stent-graft implantation for hepatic arterial bleeding: a systematic review and meta-analysis

**DOI:** 10.1186/s42155-025-00608-0

**Published:** 2025-10-13

**Authors:** Sinan Deniz, Elif Öcal, Muzaffer Reha Ümütlü, Moritz Wildgruber, Jens Ricke, Max Seidensticker, Osman Öcal

**Affiliations:** 1 Department of Radiology, University Hospital, LMU Munich, Munich, Germany; 2 Department of Diagnostic and Interventional Radiology, Heidelberg University Hospital, INF 420, Heidelberg, 69120 Germany

**Keywords:** Hepatic artery, Stent-graft, Bleeding, Patency, Liver

## Abstract

**Purpose:**

The aim of this systematic review and meta-analysis was to identify the technical and clinical outcomes of stent-graft implantation in patients with hepatic artery bleeding.

**Materials and methods:**

The PubMed database was searched for publications between 2000 and March 2025 evaluating patients treated with stent-graft implantation for hepatic arterial hemorrhage. The outcome measurements were technical (successful hemostasis with stent-grafts) and clinical success (no rebleeding from hepatic arteries), early mortality, and stent-graft patency during follow-up. A modified Newcastle–Ottawa Scale (NOS) was used to assess the quality of the included publications. An individual patient data meta-analysis was performed using the chi-square test or Fischer exact test to identify predictors of stent-graft patency.

**Results:**

In total, 351 patients from 22 studies were included. The mean NOS score was 5.4 ± 0.95. Most patients (*n* = 323) had bleeding after surgery. The technical success rate of stent-graft placement was 94.3%. Patients with technical failure were managed either by surgery (*n* = 10) or coil embolization of the hepatic artery (*n* = 10). Rebleeding from hepatic arteries was seen in 24 patients with a clinical success rate of 92.7%. The early mortality rate was 15.6%.

Follow-up showed stent-graft patency in 76.5% (202 of 264) of the cases. Most of the stent-graft occlusions (51/62, 82.2%) were asymptomatic. No significant difference was seen in the rate of stent-graft patency between patients receiving acetylsalicylic acid or not, or dual antiplatelet treatment or not. Overlapping stent-grafts (*p* = 0.009) were significant risk factors for stent-graft occlusion.

**Conclusion:**

Stent-graft implantation in hepatic arterial bleeding is associated with high technical and clinical success rates. Despite the considerable rate of stent-graft occlusion during follow-up, occlusion is mostly asymptomatic, probably due to collateral development.

**Supplementary Information:**

The online version contains supplementary material available at 10.1186/s42155-025-00608-0.

## Introduction

Hepatic arterial hemorrhage is most commonly seen after hepatobiliary or pancreatic surgeries, and bleeding is usually caused by vascular erosion due to surrounding infectious collection or pancreatic fistula [1–3]. Historically, surgery has been the initial management option; however, it is associated with high morbidity and mortality, especially in patients with recent surgery [4, 5]. Although coil embolization of the hepatic artery offered a minimally invasive alternative, acute loss of arterial inflow is associated with major complications despite the secondary inflow of the liver via the portal vein. In patients with recent hepatobiliary surgery, embolization of the hepatic artery can lead to liver necrosis, and a high rate of liver failure-related mortality has been reported in such cases [6]. In patients with poor collaterals and compromised portal flow, the risk for hepatic complications increases significantly [7]. Additionally, bile ducts are supplied only by arteries and are at particular harm, especially in recent biliary anastomosis. Since the first case report of stent-graft implantation into the hepatic artery for bleeding [8], several case reports and a few case series have reported favorable outcomes with low morbidity and mortality. However, there is variability in the technical and post-procedural approaches used in these studies, including the post-procedural anticoagulation regimen. And despite the increased use of stent grafts in such settings, there is no consensus guideline endorsed by the interventional radiology communities due to the lack of clear evidence. The current study aimed to evaluate the technical and clinical outcome of stent-graft implantation for hepatic arterial bleeding and the long-term patency of the stent-grafts by conducting a systematic review and meta-analysis of the current literature.

## Materials and methods

A systematic review was performed of published literature on reported cases of hepatic arterial bleeding in which stent-grafts were used to control bleeding. Since this study was a meta-analysis, it was not subject to approval by the institutional review board.

### Literature search strategy

**Fig. 1:**
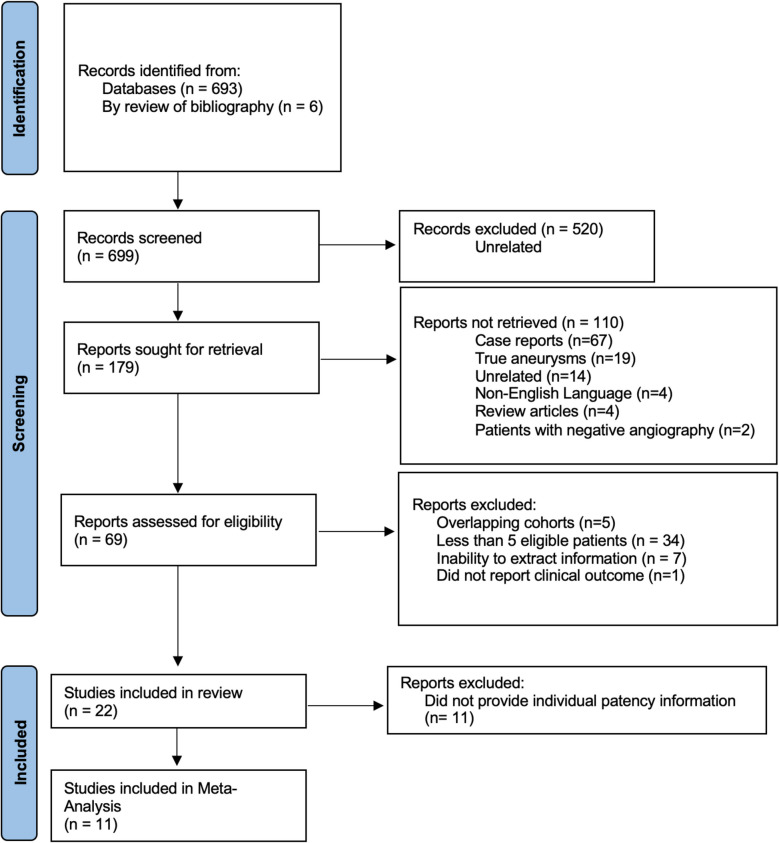
Study flow diagram

The PubMed database (including articles indexed by MEDLINE) was searched in its entirety from 2000 until March 2025 using combinations of the following keywords: “hepatic artery OR liver artery” [MeSH Unique ID: D006499], “bleeding OR hemorrhage” [D006470], “stent OR stent-graft” [D015607]. No language limit was applied for the initial search. The records were then systematically evaluated in terms of inclusion and exclusion criteria. A manual search of the references of the identified manuscripts was also performed. Two researchers independently performed the initial search and assessed the abstracts of the identified publications. The studies identified by only one researcher were discussed together for inclusion. This meta-analysis follows the PRISMA (Preferred Reporting Items for Systematic Reviews and Meta-Analyses) guidelines. The literature search strategy flowchart is presented in Fig. 1.

### Selection criteria

The inclusion criteria for this study were that the study must include patients who have undergone stent-graft implantation for bleeding or pseudoaneurysm from hepatic arterial circulation, including common hepatic artery (CHA), proper hepatic artery (PHA), right hepatic artery (RHA), left hepatic artery (LHA), or gastroduodenal artery stump (GDA). Exclusion criteria were as follows: the study did not report the clinical outcome of the patients, the study was a review paper without an original patient cohort, the study had potential overlapping cohorts (in this case, the most recent study was included), patients who underwent elective stent-grafting of a true arterial aneurysm (without rupture), and studies with less than five eligible patients with stent-graft placement into hepatic arteries for bleeding. If the study reports a cohort with bleeding from several arteries and/or mixed treatment techniques, and it was not possible to extract the information about the technical and clinical outcome of the patients with stent-graft placement into hepatic arteries separately, these studies were similarly excluded.

### Data extraction and analysis

Two independent researchers performed data extraction using a standardized form that included authors, year of publication, study design, number of patients, gender, etiology of hepatic arterial bleeding, bleeding vessel, procedural outcome, complications, and early mortality (30 days or in-hospital). The authors and their affiliations were checked to identify any potential overlapping cohorts. In studies that also included patients who were treated with other techniques (embolization, surgery) or those who had bleeding from other arteries, only the patients who underwent stent-graft placement for hepatic arterial bleeding were included in this analysis.

Primary endpoints were technical and clinical success of stent-graft implantation. Secondary endpoints were early mortality and stent-graft patency rate. Technical success was defined as the exclusion of the pseudoaneurysm or cessation of the bleeding through the stent-graft implantation. The technical success rate was calculated in a summative narrative fashion by taking the total amount of reported successful stent-graft procedures and dividing it by the total number of attempted stent-graft procedures. Clinical success was defined as no rebleeding from the hepatic arterial vasculature within 30 days. The clinical success rate was calculated by subtracting the patients with rebleeding from hepatic arterial vasculature within 30 days from the total number of reported successful stent-graft procedures. Outcomes of the patients with technical or clinical failure were separately evaluated. In addition, the patency of stent-grafts at the end of follow-up was assessed, and clinical presentation of stent-graft occlusion was recorded. From the publications included in the systematic review, studies reporting anticoagulation regimen and stent-graft patency were included in the meta-analysis. The Newcastle–Ottawa Scale (NOS) was used to assess the quality of the included publications; however, due to all the included studies being single-arm, the NOS was modified by removing 3 components that assess comparability and the control group. Quantitative synthesis of the included studies was performed in R language environment (version 3.4.1) with the “meta” package (version 4.9‐2). Stent-graft patency rates were pooled with a random effects model. Additionally, studies with individual patient and procedural information with stent-graft patency were included in the individual patient data meta-analysis. Univariable analysis of the relationship between periprocedural characteristics and stent-graft patency was assessed with the chi-square test or Fisher exact test for patients with individual data. Variables with a *p*-value of < 0.05 in the univariable analyses were analyzed in multivariable model. A two-sided *p*-value < 0.05 was considered statistically significant.

## Results

Six hundred ninety-nine articles were identified, 520 of which were deemed to be non-relevant during abstract screening. The remaining 179 articles’ full text was accessed for assessment. One hundred fifty-eight articles were excluded due to results summarized in the PRISMA flow diagram (Fig. 1).

There were 22 articles, including 351 patient cases to meet the inclusion criteria [9–30]. Twelve articles consisted of patients with hepatic arterial bleeding managed with stent-graft placement, four articles included patients with hepatic arterial bleeding managed with different techniques (in addition to stent-graft implantation), four articles included patients who underwent stent-graft placement for bleedings from several arteries (in addition to hepatic arteries), and two articles on several endovascular techniques (in addition to stent-graft implantation) for bleedings after pancreatic surgeries. However, only the patients with stent-graft implantation in the hepatic arteries were considered in this study. All studies were retrospective cohorts. The mean NOS score was 5.4 ± 0.95 (out of 6) (Table S1).

Baseline characteristics are summarized in Table 1. Two hundred forty patients were male, 67 were female, and in 44 patients, gender was not specified. Most cases had bleeding after surgery (*n* = 323), being pancreatic surgery in 260, hepatobiliary resection in 24, liver transplantation in 9, cholecystectomy in 1, duodenal operation in 1, gastrectomy in 1, and not specified in 27 cases. The etiology was trauma in three patients, ERCP in two, tumor erosion in two, chemotherapy in one, alpha-1-antitrypsin deficiency in one, and idiopathic in one. Etiology was not specified in 18 cases.

**Table 1:** Baseline characteristics

	*n*=351
Gender	
Male	240
Female	67
n.s.	44
Bleeding Etiology	
Surgery	323
Pancreatic surgery	260
Liver resection	24
Liver transplantation	9
Cholecystectomy	1
Duodenal operation	1
Gastrectomy	1
n.s.	27
Trauma	3
ERCP	2
Tumor	2
Chemotherapy	1
Alpha-1-antitrypsin deficiency	1
Idiopathic	1
n.s.	18
Bleeding vessel	
GDA stump	176
CHA	71
PHA	35
RHA	15
LHA	4
Cystic artery origin	1
PHA&GDA	1
GDA&CHA	1
RHA&PHA	1
CHA&PHA	1
RHA&CHA	1
RHA&LHA	1
n.s.	26

The most common location of bleeding was GDA stump with 176 patients. The bleeding artery was CHA in 71, PHA in 35, RHA in 15, PHA/CHA in 5, LHA in 4, the cystic artery origin in 1, PHA&GDA in 1, GDA&CHA in 1, RHA&PHA in 1, CHA&PHA in 1, RHA&CHA in 1, and RHA&LHA in 1 patient. The exact location of bleeding was not specified in 26 patients and given as hepatic artery.

### Technical success

Outcome of the patients included in this systematic review is summarized in Table 2. Technical success was achieved in 331 out of 351 patients (technical success rate of 94.3%), including one patient with a pseudoaneurysm in whom the first session failed (due to tortuous anatomy) and successful treatment the day after.

**Table 2:** Number and outcome of the patients included in this systematic review

Author/year	No. of pts	Technical success	Clinical success^**^	Mortality	Patency
*Stoupis 07*	5	5/5	5/5	3/5	5/5
*Goltz 10*	8	8/8	7/8	3/8	8/8
Wang 10	9	9/9	7/9	3/9	6/6
*Boufi 11*	8	6/8	6/6	2/8	6/6
Künzle 13	6	5/6	4/5	2/6	n.s
Lü 13	8	6/8	6/6	0/6^#^	6/6
Bellemann 14*	24	21/24	19/21	5/24	*2 thrombosis*
*Lim 14*	17	16/17	16/16	3/17	12/16
Huo 15	8	8/8	8/8	0/8	n.s
*Hassold 16*	14	13/14	12/13	3/14	7/12
*Venturini 17*	18	18/18	14/18	3/18	10/11
Muglia 20	5	4/5	4/4	0/5	n.s
*Shiari 20*	6	6/6	6/6	0/6	3/6
Cui 20	17	17/17	13/17	4/17	13/13
*Öcal 21*	27	25/27	23/25	8/27	10/22
*Kamada 21*	9	8/9	6/8	2/9	8/8
Pedersoli 21	25	24/25 (1 in second)	23/24	7/25	9/16
Watanabe 22	20	20/20	20/20	0/20	13/20
Min 22	21	20/21	20/20	0/20^#^	10/20
Okumura 24	11	11/11	11/11	0/11	9/11
Kim 24	61	59/61	57/59	3/61	49/59
Li 25	24	22/24	20/22	3/22^#^	18/19

Italic type indicates the studies included in the meta-analysis

^*^Only reported stent-graft occlusions; no information was provided on the patency of the rest

^**^The patients with bleeding from vessels other than hepatic arteries are not considered

^#^The outcome of patients with technical failure is not reported

While ten patients with technical failure were managed surgically, the hepatic artery was embolized with coils in ten cases. Reasons for technical failure were rupture of the hepatic artery in three, dissection of celiac truncus in two, stenosis preventing stent-graft implantation in one, tortuosity and stenosis of CHA in one, vessel tortuosity in two, inability to catheterize the hepatic artery in two, persistent endoleak in two, stent migration in one, immediate occlusion of stent-graft in one, severe spasm in one, severe kinking in one, insufficient proximal landing zone in one, and multiple pseudoaneurysms in PHA and HCA in one patient. The reason was not specified in one patient who underwent embolization of the hepatic artery with coils immediately after stent-graft implantation.

### Clinical success

Clinical success was achieved in 307 out of 331 patients with successful stent-graft implantation (clinical success rate of 92.7%). Re-bleeding was seen in 24 patients 1 h to 30 days after the procedure. Five of these patients died without any further interventions. Six patients underwent transcatheter embolization; three of them died, two not specified. Five patients underwent surgical management, including one patient with negative angiography, and all of these five patients were lost during the hospital stay. One patient was treated with intermittent balloon occlusion to preserve the patency of the hepatic arterial vasculature but did not survive. Seven patients were managed with additional stent-graft implantation (four survived, one not specified).

Early mortality was seen in 54 (15.6%) patients out of 346 patients with attempted stent-graft implantation (this information was not provided in five patients with technical failure). The most common reason for mortality was multiorgan failure, with 33 patients. In 10 patients, the leading cause was sepsis, uncontrolled bleeding in 4, renal failure in 2, and disseminated intravascular coagulation, liver failure, cardiogenic shock, and cerebral death each in one patient. Out of 23 patients with imaging follow-up before death, stent-grafts were patent in 18 (78.2%).

### Stent-graft patency

In 18 studies, the results of imaging follow-up in terms of stent-graft patency were given (Table 3). The stent-graft patency rate was 76.5% (202 out of 264 patients). Stent-graft thrombosis was seen 1 to 2407 days after the procedure. In 51 of 62 patients with occluded stent grafts, thrombosis was asymptomatic. Five of the rest of the eleven patients developed liver abscesses; three had liver infarction, one had segmental perfusion defects, and one developed biloma. The other patient was lost 8 days after the stent-graft thrombosis; however, the patient had already had multiorgan failure before the stent-graft occlusion. While three studies did not mention the patency of stent-grafts, one study reported two stent-graft thromboses. The patient with stent-graft occlusion 2 days after the procedure developed liver necrosis and died of sepsis. The other patient with thrombosis on day 16 was successfully treated with thrombolysis.

**Table 3:** Stent-graft patency rate and outcome of stent-graft thrombosis

Author/year	Antiplatelet regimen	Patency	Number of stent occlusion	Timepoint of stent occlusion	Outcome
Stoupis 07	n.s	5/5	0		
Goltz 10	5000 IU Heparin (in case of pseudoaneurysm) + ASA lifelong	8/8	0		
Wang 10	ASA 100 mg after discharge	6/6	0		
Boufi 11	No standard regimen dAPT for 3–6 months for stable patients	6/6	0		
Künzle 13	ASA lifelong	n.s	n.s		
Lü 13	dAPT 12 weeks + ASA lifelong	6/6	0		
Bellemann 14*	2–3 days heparinization + dAPT 4–6 weeks + ASS lifelong	2 thrombosis	2	2 days 16 days	Liver necrosis, sepsis, exitus Underwent thrombolysis
Lim 14	No standard regimen dAPT (9/17)	12/16	4	1 day 1 day 30 days 182 days	Liver infarct (both lobes), exitus Liver infarct (right lobe), exitus Asymptomatic Segmental perfusion defect
Huo 15	n.s	n.s	n.s		
Hassold 16	No anticoagulation or antiplatelet	7/12	5	22 days 59 days 78 days 230 days 571 days	Liver abscess Asymptomatic Asymptomatic Asymptomatic Asymptomatic
Venturini 17	dAPT for 6 months + ASA lifelong	10/11	1	7 months	Asymptomatic
Muglia 20	n.s	n.s	n.s		
Shiari 20	3000 IU Heparin + no standard regimen (n.s.)	3/6	3	3 days 2.9 months 4.1 months	Asymptomatic Liver abscess Asymptomatic
Cui 20	No anticoagulation or antiplatelet	13/13	0		
Öcal 21	No standard regimen ASA 8/22, dAPT 4/22 ASA + enoxaparin 7/22, dAPT + enoxaparin 2/22, enoxaparin 1/22	10/22	12	1 days 3 days 5 days 16 days 20 days 24 days 25 days 45 days 150 days 210 days 360 days 390 days	Asymptomatic Liver abscess Asymptomatic Asymptomatic Asymptomatic Asymptomatic Multiorgan failure, Exitus Asymptomatic Asymptomatic Liver abscess Asymptomatic Asymptomatic
Kamada 21	No standard regimen	8/8	0		
Pedersoli 21	24 h heparinization + dAPT 6 weeks + ASA lifelong +	9/16	7	n.s	6/7 Asymptomatic 1/7 Liver abscess
Watanabe 22	dAPT for 12months	13/20	7	3 months 3 months 3 months 6 months 6 months 6 months 24 months	Asymptomatic Asymptomatic Asymptomatic Asymptomatic Asymptomatic Asymptomatic Asymptomatic
Min 22	No standard regimen. 6 months antiplatelet (n.s.) in 7 patients	10/20	10	7 within 1 year, 3 in average of 891-day	All asymptomatic
Okumura 24	dAPT for 6 months	9/11	2	1 month 1 month	Asymptomatic Asymptomatic
Kim 24	No anticoagulation/antiplatelet	49/59	10	8 days 28 days 5–2407 days	Liver infarction Biloma Asymptomatic (8 patients)
Li 25	No antiplatelet	18/19	1	6 days	Asymptomatic

*ASA* acetylsalicylic acid, *n.s.* not specified

^*^Only reported stent-graft occlusions; no information was provided on the patency of the rest

Individual follow-up imaging duration was given in nine studies with 101 patients. The median follow-up duration was 150 days in all patients, 210 days in patients without in-hospital mortality, and 300 days (range, 1–2521) in patients with patent stent-grafts. Three studies reported the mean follow-up duration (315, 420, 690 days), one study reported median and interquartile range (63, 10–396 days), two studies reported median duration to stent-graft occlusion (78 and 148 days), one study reported all occlusions were beyond 3 months of implantation, and one study reported seven occlusions within 1 year and the rest three with a median duration of 891 days.

### Anticoagulation/antiplatelet regimen

Three studies (17 patients) did not report anticoagulation information, while three studies (20 patients) employed a non-standardized regimen and did not provide details. In three studies (89 patients), no anticoagulation was given; lifelong acetylsalicylic acid (ASA) was given in three studies (22 patients), and dual antiplatelet treatment (dAPT) in six studies (104 patients). Four studies employed a non-standardized regimen, and dAPT was given in 9 of 17 patients, 7 of 20 patients, and 6 of 25 patients (Table 3).

In total, for 14 studies, anticoagulation and stent-graft patency information were available. There was no significant difference in the rate of stent-graft patency between patients receiving ASA or not (72.85% vs. 83.7%, *p* = 0.066), or dAPT or not (73.4% vs. 80.8%, *p* = 0.264).

### Individual patient data meta-analysis

Individual patient data meta-analysis included 142 patients from eleven articles, with patent stent-grafts at the end of follow-up in 96 patients and 28 stent-graft occlusions. Univariable analysis identified overlapping stent-grafts (*p* = 0.001), stents placed into RHA or LHA (*p* = 0.004), and balloon-expandable stent-grafts (*p* = 0.038) as significant risk factors for stent-graft occlusion (Table 4). Overlapping stent-grafts was the only risk factor for stent occlusion in the multivariable analysis (*p* = 0.022).

**Table 4:** Univariable and multivariable analysis of risk factors for stent-graft occlusion

Variable	Patency		*p*-value	Multivariable
	**Patent** ***N* = 96 (77.4%)**	**Occluded** ***N* = 28 (22.6%)**		***p*-value**
**Malignancy**	37/53 (69.8%)	17/19 (89.5%)	0.13	
**Etiology (surgery)**	72/72 (100%)	21/23 (91.3%)	0.057	
**Gender (male)**	59/71 (83.1%)	22/24 (91.7%)	0.5	
**Pseudoaneurysm**	34/57 (59.6%)	13/21 (61.9%)	0.9	
**Overlapping stent-grafts**	15/90 (16.6%)	13/28 (46.4%)	**0.001**	**0.022**
**Dual antiplatelet**	38/93 (40.8%)	7/25 (28%)	0.2	
**Stented vessel**			**0.004**	0.103
*Proximal (CHA, PHA, GDA)*	75/84 (89.2%)	15/24 (62.5%)		
**Stent size ≥ 5 mm**	70/90 (77.7%)	17/28 (60.7%)	0.073	
**Self-expandable stent-grafts**	70/95 (73.7%)	13/26 (50%)	**0.038**	0.136

Data presented as numbers and percentages. Patients with missing information were removed from each line separately

Bold type indicates statistical significance

## Discussion

Stent-graft implantation in patients with bleeding from hepatic arteries is associated with high technical success (94.3%) and clinical success (92.7%) rates. However, early mortality rates are relatively high (15.6%), and there is a relevant risk for stent-graft occlusion during follow-up. Stent-graft occlusions are mostly incidental, and patients with overlapping stent-grafts are at increased risk for thrombosis.

Hepatic arterial bleeding is mostly encountered as a post-operative complication. In 92% of patients identified in this analysis, the etiology was a recent surgery. Although it is a rare post-operative complication, hepatic arterial bleeding is significantly associated with worse outcomes. In patients with post-operative hemorrhage after pancreaticoduodenectomy, the mortality rate increases to 24.9% compared to 4.0% in patients without bleeding [31]. Hepatic arterial bleeding poses an additional challenge to achieve hemostasis while preserving the hepatic arterial flow. Watanabe et al. showed that hepatic arterial bleedings managed with stent graft implantation are associated with significantly lower major hepatic complications as well as in-hospital mortality compared to embolization of hepatic arteries [30]. With advancements in catheter and delivery system technologies, stent-graft implantation has been used in patients with bleeding from visceral arteries [32]. This systematic review showed the technical success rate of stent-graft implantation for hepatic arterial bleeding was 94.3%. Reasons for technical failure were various, but considering patients with rupture or dissection of the artery during the procedure, the development of lower profile stent-graft delivery systems with improved navigability could enhance the technical outcome further. However, all of the included studies were retrospective, and underreporting of failed cases should also be considered.

In patients with technical success (with successful stent-graft implantation, *n* = 331), a rebleeding was seen in 24 patients (clinical failure rate 7.3%), and 16 (66.6%) of these 24 patients were lost during the hospital stay (not specified for three patients). In patients with surrounding infectious collections or pancreatic fistula, continuing vascular erosion could lead to rebleeding. Li et al. found the presence of pancreatic fistula as a risk factor for rebleeding [27]. Furthermore, in a cohort of 1669 patients with pancreatectomy, 12 of 14 patients who died due to bleeding complications had accompanying pancreatic fistula [3]. However, this situation was not assessed in most of the articles evaluated in this analysis. Other identified risk factors for rebleeding were intensive-care patients, the presence of shock, and smaller stent-grafts [22, 26]. Considering the importance of correct stent-graft size in sealing the bleeding point, spasm due to vascular injury before stent-graft implantation could mislead to a selection of smaller stent-grafts which might lead to rebleeding in a few days after resolution of spasm with hemostasis, which was the case in some of the rebleeding cases and treated with additional stent-grafts. The early mortality rate after attempted or successful stent-graft implantation was 15.6%. Most deaths were caused by multiorgan failure or sepsis, representing the critical condition of those patients with postsurgical hemorrhage. Stent-grafts were patent in 78.2% of patients who were lost after stent-graft implantation and received imaging, which precludes the potential connection between stent-graft occlusion and mortality.

The stent-graft patency rate was 76.5% in patients with available follow-up imaging. In 82.2% of the cases with stent-graft occlusion, the thrombosis was diagnosed incidentally. Despite the considerable occlusion rate during follow-up, with immediate hemostasis, stent-grafts offer time to treat the underlying medical condition of the patient. Additionally, Lim et al. showed that stent-graft occlusion happens gradually from partial thrombosis to complete occlusion [33]. Lack of openings between stent struts in stent-grafts delays the endothelization, and patients with incomplete platelet inhibition could present with late thrombosis [34, 35]. This situation was reflected in the increased risk of thrombosis in patients with overlapping stent-grafts. Besides this, stent-graft occlusion risk was higher in stent-grafts placed in RHA or LHA or in balloon-expandable stent-grafts, although they were not significant in the multivariable analysis. A higher risk of occlusion in stent-grafts placed in RHA or LHA has already been reported [22]. The correlation between stent-graft occlusion and balloon-expandable stent-grafts was not significant in multivariable analysis, and the significant correlation in the univariable analysis was probable due to a higher rate of overlapping stent-grafts and placement in RHA or LHA in balloon-expandable stent-grafts than self-expandable grafts (29% vs. 16% and 25% vs. 11%). This meta-analysis did not identify a statistically significant difference in stent-graft thrombosis risk in patients receiving ASS or dAPT compared to patients who did not receive these treatments. There is no consensus on the antiplatelet regimen in patients who received stent-graft implantation due to bleeding, and the antiplatelet and follow-up regimen varied between included studies. Considering most patients had pancreatic surgery, local factors such as accompanying pancreatic fistula could be a potential reason for the high thrombosis rate in patients receiving antiplatelet agents. Despite the clear lack of benefit from antiplatelet agents, considering the importance of liver artery patency, we support the antiplatelet treatment in those patients after achieving hemostasis. Additionally, the variations in follow-up imaging protocols and durations have to be considered.

This study has limitations that are inherent to a systematic review. Some of the identified studies could not be included because required outcome parameters were not reported. All of the included studies were retrospective, and interventional techniques, anticoagulation/antiplatelet treatment regimens, and follow-up intervals were not standardized. Additionally, patient compliance was not considered in those studies. No prospective or randomized controlled study has been identified on this subject. This situation limits the value of conclusions drawn from this meta-analysis. However, this study summarizes the current situation of the literature. Furthermore, due to the small number of events, no meta-analysis could have been performed for the outcomes of technical success, clinical success, or early mortality. Moreover, since all studies were single-center and most used a sole antiplatelet approach (either none or the same for all patients) and did not report any cases of stent-graft occlusion, a meta-analysis of all included studies evaluating the correlation between antiplatelet regimens and stent-graft occlusion would have required a Bayesian correction to address rare events for a majority of the patients, which would be misleading due to the low number of patients. Additionally, due to the lack of such analysis of all included studies, the heterogeneity of included studies could not be statistically explored. To overcome this limitation, an individual patient data meta-analysis has been performed using patient characteristics for each patient in studies providing these information, which is limited due to the lack of missing data. Due to the rarity and emergency of bleeding from the hepatic arteries, it is difficult to perform a randomized trial on this topic; however, a prospective multicenter registry could overcome these limitations.

In conclusion, hepatic arterial bleeding can be managed with stent-graft implantation with high technical and clinical success rates. However, early mortality is not uncommon, primarily due to the underlying critical condition of the patients. Although it is usually asymptomatic, there is a considerable risk for stent-graft occlusion during follow-up.

## Supplementary Information

Additional file 1: Table 1.

## Data Availability

The datasets used and/or analyzed during the current study are available from the corresponding author on reasonable request.
