# Supplementary material for: Stent-graft implantation for hepatic arterial bleeding: a systematic review and meta-analysis
Source: CVIR Endovasc. 2025 Oct 13;8:80. doi: 10.1186/s42155-025-00608-0 (PMC12518196; doi:10.1186/s42155-025-00608-0)
Supplement: Supplementary file 1 — Additional file 1: Table 1. [file 42155_2025_608_MOESM1_ESM.pdf]

Table S1. Newcastle–Ottawa Scale for assessment of quality of included cohort studies.

Study	Selection			Outcome			**Total quality score**
Quality assessment criteria	Representativeness of cases (All relevant cases during study period)	Ascertainment of exposure (Information on anticoagulation)	Demonstration that outcome of interest was not present at start of study (patency of hepatic artery)	Assessment of outcome (assessment of stent-graft patency)	Follow–up Length (less than 3 months)	Loss to follow–up rate (less than 15% for stent-graft patency)	
*Stoupis 07**	★		★	★		★	4
Goltz 10	★	★	★	★	★	★	6
Wang 10	★	★	★	★	★	★	6
*Boufi 11**	★	★	★	★	★	★	6
Künzle 13	★	★	★				3
Lü 13	★	★	★	★	★	★	6
Bellemann 14*	★	★	★		★		4
Lim 14	★	★	★	★	★	★	6
Huo 15*	★		★	★	★		4
Hassold 16	★	★	★	★	★	★	6
Venturini 17	★	★	★	★	★	★	6
Muglia 20*	★		★	★	★		4
*Shiari 20**	★	★	★	★	★	★	6
Cui 20	★	★	★	★	★	★	6

Öcal 21	★	★	★	★	★	★	6
*Kamada 21**	★	★	★	★	★	★	6
Pedersoli 21	★	★	★	★		★	5
Watanabe 22	★	★	★	★	★	★	6
Min 22*	★	★	★	★	★	★	6
Okumura 24	★	★	★	★		★	5
Kim 24	★	★	★	★	★	★	6
Li 25	★	★	★	★	★	★	6

Mean Score: 5.4±0.95

Newcastle−Ottawa Scale is modified for single-arm studies.
